# Increased Thalamic Gamma Band Activity Correlates with Symptom Relief following Deep Brain Stimulation in Humans with Tourette’s Syndrome

**DOI:** 10.1371/journal.pone.0044215

**Published:** 2012-09-06

**Authors:** Nicholas Maling, Rowshanak Hashemiyoon, Kelly D. Foote, Michael S. Okun, Justin C. Sanchez

**Affiliations:** 1 Department of Neuroscience, University of Florida, Gainesville, Florida, United States of America; 2 Department of Biomedical Engineering, University of Miami, Coral Gables, Florida, United States of America; 3 Department of Neurosurgery, University of Florida, Gainesville, Florida, United States of America; 4 Center for Movement Disorders & Neurorestoration, University of Florida, Gainesville, Florida, United States of America; 5 Department of Neurology, University of Florida, Gainesville, Florida, United States of America; 6 Neuroscience Program, University of Miami, Miami, Florida, United States of America; 7 Miami Project to Cure Paralysis, University of Miami, Miami, Florida, United States of America; University of Adelaide, Australia

## Abstract

Tourette syndrome (TS) is an idiopathic, childhood-onset neuropsychiatric disorder, which is marked by persistent multiple motor and phonic tics. The disorder is highly disruptive and in some cases completely debilitating. For those with severe, treatment-refractory TS, deep brain stimulation (DBS) has emerged as a possible option, although its mechanism of action is not fully understood. We performed a longitudinal study of the effects of DBS on TS symptomatology while concomitantly examining neurophysiological dynamics. We present the first report of the clinical correlation between the presence of gamma band activity and decreased tic severity. Local field potential recordings from five subjects implanted in the centromedian nucleus (CM) of the thalamus revealed a temporal correlation between the power of gamma band activity and the clinical metrics of symptomatology as measured by the Yale Global Tic Severity Scale and the Modified Rush Tic Rating Scale. Additional studies utilizing short-term stimulation also produced increases in gamma power. Our results suggest that modulation of gamma band activity in both long-term and short-term DBS of the CM is a key factor in mitigating the pathophysiology associated with TS.

## Introduction

Tourette syndrome (TS) is a complex neuropsychiatric disorder characterized by the childhood onset of multiple motor and phonic tics [1], [2]. It affects an estimated 1% of the population [3] and is marked by high comorbidities (80–90%), including obsessive compulsive disorder (OCD), attention deficit hyperactivity disorder (ADHD), anxiety, behavioral disorders, and depression [4]–[9]. While recent work in molecular biology and neuroimaging has provided new insights into the pathophysiology of TS, there remain formidable gaps in understanding tic etiology [2], [10]–[12].

Although the majority of tic manifestations remit by adulthood [1], [2], one third of patients continue to experience tic burden that may become severe or even malignant [13], [14]. These symptoms can be extremely disruptive, socially inappropriate, and resistant to traditional therapies [15]–[17]. Historically, such cases were treated with ablative brain lesions in the centromedian nucleus (CM) of the thalamus or globus pallidus internus (GP_i_) [18], [19]. While the mechanism of action of deep brain stimulation (DBS) remains unclear, its therapeutic potential is now being tested in both of these targets for treatment-refractory TS [16], [17], [20]–[25]. Results from early studies suggest DBS of the CM may be a promising alternative for more severe cases [17], [26]. To this end, acute microelectrode recordings and chronic macroelectrode recordings from implanted leads will enable the elucidation of not only the neural correlates of symptomatology, but also of the dynamic changes that occur over time with respect to DBS therapy.

The success of surgical treatments in the CM thalamus support the notion that this region likely contributes to a network dysfunction responsible for tic generation [19]. The CM is an intralaminar thalamic nucleus that has reciprocal connections with the action-gating pathways of the basal ganglia [27], [28]. This region has been demonstrated to process relevant sensory, executive, and arousal information that is important for motor planning [29]–[32]. Because of its neuroanatomical connectivity, the CM thalamus is uniquely positioned to affect the neural circuitry underlying TS symptomatology. Neuronal population activity producing tic phenomenology is likely encoded by network oscillations and their dynamics. For example, the presence of gamma band oscillations reflects a state of high neuronal excitability and synchronization by cell ensembles which are critical for neuronal communication and normal brain function [33]–[36]. Abnormal gamma oscillations have been implicated in such disorders as Parkinson’s disease, schizophrenia, and depression [37]–[39]. However, very little is known about the functional significance of these oscillations in TS, aberrant or otherwise, or how they are modulated by DBS therapy [40]. Our work directly addresses this issue by providing the first evidence for the possible functional role of brain oscillations in the pathophysiology of TS.

In this study, we describe the electrophysiology and clinical benefit of DBS in five human TS subjects implanted with Neuropace™ neurostimulators (Neuropace, MountainView CA) [41], [42]. This novel experimental and clinical paradigm enabled the capture of LFPs from the CM thalamic region and their relationship to tic expression over the course of six months, thus providing long-term study of thalamic neurophysiology in human TS subjects undergoing DBS. We utilized this electrophysiology coupled with tic phenomenology to correlate chronic, temporal changes of neural activity with clinical evaluations of tic expression.

## Materials and Methods

### Surgery & Subjects

Five out of nine subjects screened met the DSM-IV-TR criteria for TS as well as the criteria for TS DBS proposed by the Tourette Syndrome Association [43], [44] and were enrolled in this study (FDA clinical trial NCT01329198 (clinicaltrials.gov)). Three females and two males between 28–39 (mean 34.4) years of age all had severe motor and phonic tics with disease duration at the time of implantation of 20–37 (mean 28.8) years (Table 1). Tic severity was assessed at screening, before surgery, and at each subsequent visit using the Yale Global Tic Severity Scale (YGTSS) and the Modified Rush Tic Rating Scale (MRTRS). All subjects had comorbidities with OCD symptoms. All subjects were refractory to traditional medications and were approved as candidates for DBS surgery by an interdisciplinary expert panel and an independent ethics panel. All procedures were approved by the University of Florida Institutional Review Board, and all subjects provided informed consent.

**Table 1:** Subject demographics.

ImplantNumber	Sex	Age	DiseaseDuration	Common Tics	BehavioralComorbidities
TS1	F	34	26	Head jerks, limb-jerking, slapping/hitting self and hittingnearby objects, abdominal-tensing, coprolalia	OCD moderate and chronic
TS2	M	37	34	Eye-rolling, rotating wrists and shoulders, cracking joints,hitting nearby objects, vomiting	ADHD hyperactive and impulsive, stable and secondary substance dependency, OCD traits
TS3	M	28	20	Face-scrunching, arm jerks, head twists, bending at thewaist, copropraxia, squawking, grunting, sniffing	OCD, moderate and chronic
TS4	F	39	37	Eye-rolling, jaw cracking, head twists, fingertip tapping,hits with elbow, copropraxia, growling, coprolalia	OCD mild to moderate and chronic, PTSD mild and chronic (resolved at time of DBS)
TS5	F	36	27	Fingertip waving, grimacing, eye-rolling, echolalia, yelling, growling	OCD current moderate and chronic, PTSD (past, resolved), MDD (past, resolved)

Components from this table have been reproduced with permission from Okun (Archives of Neurology Express, 2012) and have been published with the original NIH supported FDA clinical trial (clinicaltrials.gov).

To implant DBS electrodes into the CM thalamus, stereotaxic neurosurgery was performed. Localization of the CM thalamus was achieved by the use of a 3T MRI scan, an FGATIR MRI protocol [45], and a morphable superimposed atlas. Intra-operative microelectrode recordings were performed to assess and confirm the target location by identifying characteristic neuronal firing patterns along the electrode trajectory as well as ‘border cells’ at the target. The microelectrodes (FHC, Bowdoin, ME) were advanced to the target using a micropositioner (FHC, Bowdoin, ME) and electrophysiological recordings were collected in real-time. Once the optimal implantation area was established, the DBS macroelectrode was inserted and the Neuropace neurostimulator was connected and fixed in the cranium. Test LFPs were recorded in the operating room following internalization of the leads to evaluate functionality of the implanted hardware. Additional methodological detail on targeting (Figure S1, Table S1) and signal analysis can be found in the Appendix S1.

### Hardware and Stimulation

Three of the five subjects enrolled in the study received two unilateral pulse generators each controlling ipsilateral leads. One subject (TS1) initially received a single generator controlling bilateral leads, but after four months had two replacement generators implanted, each serving unilateral leads. One subject (TS2) received a single generator serving bilateral leads for the duration of the study. Local field potentials were recorded from macroelectrodes with cylindrical contacts (1.27 mm diameter, 2.1 mm length, 3.5 mm center to center spacing, average impedance 500–600 Ω) implanted in the CM region.

Subjects were randomized for initial post-operative stimulation onset. Thus, TS1 and TS3 received stimulation beginning with the first visit, one month post-operatively, and TS2, TS4, and TS5 started stimulation at the second visit (i.e. two months post-operatively). Stimulation was delivered by alternating brief periods of on and off stimulation conditions (scheduled stimulation paradigm). Settings, including lead polarity, current, pulse width, frequency, and duty cycle were customized at each visit for each subject. Stimulation settings among the subjects had ranges from 1–4.50 mA current, 80–240 µs pulse width, 100–149.2 Hz frequency, and 17%–50% duty cycle. All subjects were stimulated with a unipolar setting on contact 1 (the most ventral/deepest contact) with the exception of TS2 who was stimulated on contact 2. This decision was based on optimal clinical responses.

### Experimental Setup

**Figure 1:**
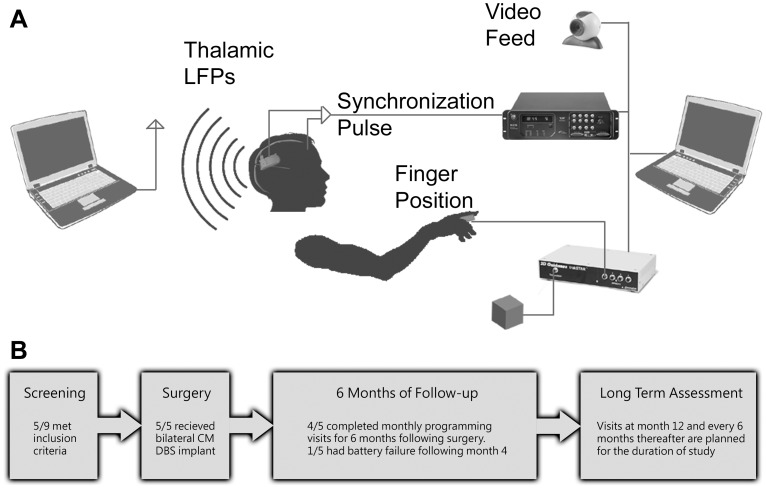
Study design. A) Experimental testbed for synchronous recording of local field potentials and tic expression. B) Data collection timeline.

A novel experimental test bed was developed to study the relationship between LFPs and tic behavior in TS subjects. The system (Figure 1A) consisted of an Ascension (Burlington, VT) Trakstar positional sensor, video recording, and the Neuropace (MountainView, CA) RNS-300 DBS device. The Neuropace DBS stimulator is capable of responsive stimulation and recording LFPs postoperatively [41], [42]. Thalamic LFPs were transmitted wirelessly from the Neuropace RNS-300 implant. The implanted processor buffers four minutes of recorded data, which was subsequently transferred via a telemetry wand connected to a computer. Telemetry delays were immaterial due time-stamp synchronization during recordings. Positional data (400.2 Hz, 32 bits of resolution), timing, and video recordings (30 Fps) were digitized and stored on a second dedicated computer. A single scalp lead was placed above the implanted pulse generator to detect timing pulses and was used only to synchronize the behavioral system with physiological recordings.

Follow-up assessments consisted of monthly visits to the clinic for the first six months following implant surgery (Figure 1B). Subjects TS2, TS3, and TS5 were recorded approximately monthly for six months postoperatively. Subject TS1 was recorded on or around postoperative months 1, 2, 3, and 4. Following four months of stimulation, this subject underwent a second surgery to remove the first device and implant two new devices, each serving the original ipsilateral leads. She was re-enrolled and resumed testing protocols on postoperative months 13 and 14. For her initial six month follow-up, subject TS4 was recorded postoperatively at month 1, 2, 3, and 5 due to travel difficulties. Long term follow-ups every 6–12 months are planned for all subjects.

### Data Collection

**Figure 2:**
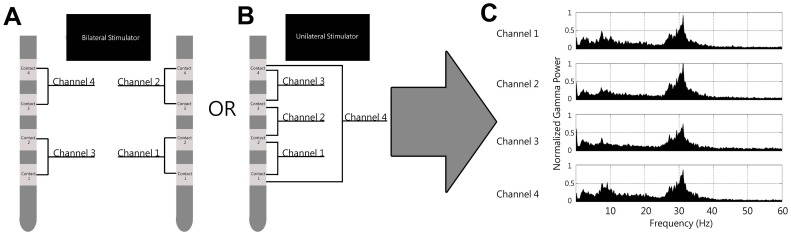
Electrode configuration and representative spectra. A) Subjects were either implanted with one stimulator controlling bilateral leads or B) two simulators each controlling one ipsilateral lead. LFPs were recorded across contact pairs as indicated. For stimulators serving bilateral leads, channels 1 & 2 represent distal and proximal LFPs in one hemisphere. Channels 3 & 4 represent distal and proximal LFPs from the opposite hemisphere. For stimulators serving unilateral leads, channels 1–3 represent consecutive contact pairs. Channel 4 represents a wider field LFP between contacts 1 & 4. C) Representative spectra from four channels recorded from a unilateral stimulator are shown.

All LFP recordings were performed while the stimulator was in the off state. Four channels of thalamic LFPs from each device were filtered (3–125 Hz, 12dB/oct) and digitized at 250 Hz from two contact pairs. For pulse generators serving unilateral leads (Figure 2A), channel 1 represents LFP recordings across contacts 1 & 2, channel 2 represents contacts 2 & 3, channel 3 represents contacts 3 & 4, and channel 4 represents contacts 1 & 4. For pulse generators serving bilateral leads (Figure 2B), channels 1 and 2 represent the LFP recordings across contacts 1 & 2 and 3 & 4 respectively, while channels 3 and 4 represent the LFP recordings from contacts 1 & 2 and 3 & 4 respectively in the contralateral hemisphere.

Recordings were made during several conditions. For the baseline condition, subjects were recorded with the stimulator off and while resting comfortably and behaving naturally. They were instructed to not suppress the urge to tic so that tic expression during baseline was unimpeded. As such, baseline consisted of periods, which were tic free in combination with periods of tic expression. These recordings were taken at each visit and typically lasted for a period of two minutes and did not exceed four minutes. This condition was the basis for all analyses except for the acute stimulation condition.

To study the short-term effects of DBS on the activity in the CM, an acute stimulation paradigm was designed in which recordings were taken immediately pre- and post-stimulation. The recordings for the condition were divided into three epochs: the first consisted of 60 seconds of baseline (as described above) recording, followed by 30 seconds of unilaterally delivered continuous stimulation (no recording), followed by another 60 second baseline recording. After a one-minute break, the condition was repeated. The entire recording protocol was then iterated in the opposite hemisphere, thus providing two sets of recordings (four total) per hemisphere per day. Acute stimulation parameters were matched to the scheduled stimulation parameters that were optimized on the previous month for each subject. Recordings for the acute stimulation condition were taken each day for two consecutive days. Spectral differences were then calculated between the pre- and post-stimulation recordings.

### Clinical Assessment

The Yale Global Tic Severity Scale (YGTSS) [46] was used to clinically assess subjects on the first day of each of their monthly follow-up visits and the outcomes recorded represented steady states of motor and vocal tic manifestations. The YGTSS represents the tic activity as reported over several weeks. In addition to the overall score, all subscores of the YGTSS for severity and impairment were evaluated with respect to neurophysiological changes.

The Modified Rush Tic Rating Scale (MRTRS) [47] was used as the clinical metric for more acute responses to changing stimulation parameters. It was typically evaluated twice daily on each study visit (before and after stimulation parameters were adjusted) to provide continuing information about meaningful changes in the clinical state. In some cases, only one scale per day was available. Measured MRTRS scores were in good agreement with YGTSS scores (Spearman’s correlation coefficient = 0.68) and consistent with the literature [47].

## Results

### Characterization of Power Spectral Density

**Figure 3:**
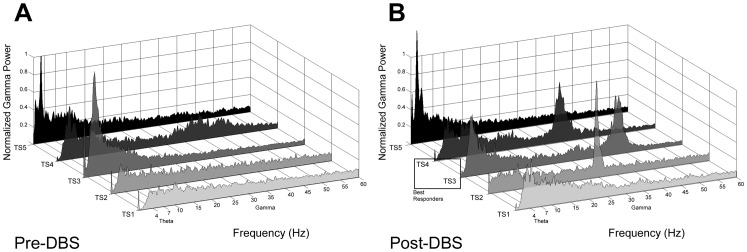
Changes in band specific power following DBS. A) Representative power spectra from all subjects in month 1 post-operatively. Recordings were taken before DBS was administered. B) Power spectra from all subjects late in the study (Months post-op for TS1 = 14, TS2 & TS4 = 5, TS3 and TS5 = 6). Months were chosen to represent the maximum gamma power. Changes in frequencies after prolonged DBS therapy are most prominent in the gamma range. All recordings taken from channel 4 (across contacts 1 and 4) in the right hemisphere in subjects with two unilateral devices or channel 1 (across contacts 1 and 2) in subjects with a single bilateral device.

Spectral analysis revealed a continuum of multiple oscillatory rhythms observed across a broad range of frequencies. Decreasing power was observed with increasing frequency as predicted by power law of natural dynamical systems. Initial comparison of the effect of pre- and post DBS on the dynamics of these frequencies indicated fluctuations in gamma band power that were not readily observed in other frequency ranges (Figure 3). These observations motivated further analysis on gamma band activity. Changes in gamma power were observed predominantly between 30–40 Hz, with a range varying between 25–45 Hz. We additionally investigated and quantified fluctuations in theta band (4–7 Hz) activity, but observed smaller and less consistent changes in power.

**Figure 4:**
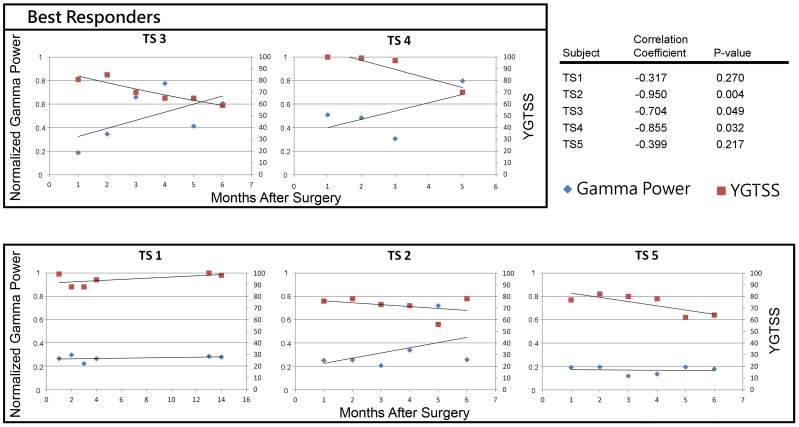
Relationship between increases in gamma power and clinical benefit for 5 subjects. Subjects TS2, TS3, and TS4 showed the best correlations. Box indicates TS 3 and TS 4 as best responders to therapy. Subject TS2 showed a spike in gamma power coincident with a drop in YGTSS. TS3 and TS4 showed a relatively monotonic increase in gamma power correlated with decreasing YGTSS. Subject TS 5 showed only a modest benefit from DBS therapy with correspondingly low increase in gamma power. All neural data shown is recorded from the right hemisphere.

### Correlation of Gamma with Clinical Improvement

Notable increases in normalized gamma band power were indicative of clinical benefit (N = 5/5). Correlation analysis revealed that the power of the gamma oscillations was inversely related to the overall severity of TS symptoms as measured by the YGTSS (Figure 4). Improvement in YGTSS was observed and compared with pre-operative screening scores. Over the six month study, all subjects showed some clinical benefit from DBS with most subjects showing a strong reciprocal increase in gamma band presence. Subjects TS2, TS3, and TS4 showed the largest negative correlations. TS1 and TS5 showed low correlations and small clinical benefits. TS3 and TS4 were the best responders to therapy (33% & 32% decrease in YGTSS) with substantially greater clinical benefit than TS1 and TS5 (1% & 18% decrease in YGTSS) as shown in Table 2. The cohort showed a positive trend in gamma power negatively correlating with decreasing YGTSS. TS2 generally exhibited minimal responses to DBS and also marginal changes in gamma between months 1–6. For all but month five, this subject’s YGTSS scores were consistently high, indicating a low average clinical benefit (18%). In month five, however, there was a large (41%) improvement in the YGTSS corresponding to a two-fold increase in gamma in the right hemisphere. Both gamma power and YGTSS returned to previous levels the following month. TS1 and TS5 showed only a small clinical improvement corresponding to very small changes in gamma power (<10%).

**Table 2:** Changes in theta, gamma, and YGTSS over the six-month study.

		Test Subjects				
		TS1	TS2	TS3	TS4	TS5
**Theta**	Left	115%	−20%	−24%	−28%	15%
	Right	160%	48%	−1%	−9%	80%
**Gamma**	Left	6%	*869%	34%	40%	6%
	Right	−5%	*124%	670%	72%	16%
**Theta**	Left	✓		✓		
	Right	✓	✓			✓
**Gamma**	left		✓	✓	✓	
	Right		✓	✓	✓	
**YGTSS**		1%	*41%	33%	32%	18%

Note *TS2 exhibited optimal benefit and maximum gamma on month 5 (benefit at month 6 was 18% compared to pre-op value). Value (⧫) in gamma increase corresponds to robust drop in the YGTSS score for month 5.

Quantification of theta band activity did not reflect a similar correlation with symptomatology. The overall changes in YGTSS as well as in gamma and theta power are presented in Table 2. These data represent the peak power (un-normalized) averaged across all channels. While fluctuations in gamma and theta power were observed for all, only the subjects that had large increases in gamma were correlated with large reductions in tic expression. For example, TS3 and TS4 showed a 670% and 72% increase in gamma, respectively and also exhibited the best clinical outcomes. In contrast, subjects TS1 and TS5 had large increases in theta (160% & 80%, respectively) with small changes in gamma and derived a small clinical benefit. Across months 1–5, TS2 exhibited opposing changes in theta band power (−20% & 48%) and yet showed considerable increase in gamma band power (869% & 124%) between hemispheres. While the greatest change in YGTSS (41%) occurred in month 5, analysis of activity in the following month revealed a precipitous drop in both YGTSS and gamma. Interestingly, in addition to prominent increases in gamma, the two best responders also showed reciprocal decreases in theta power.

**Figure 5:**
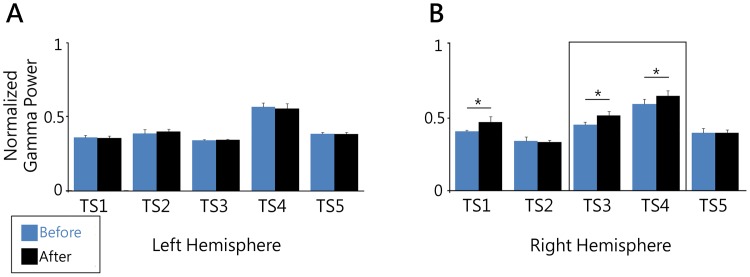
Spectral difference plots were computed to visualize changes in gamma power following acute stimulation. Power spectra for each channel were averaged for 60 seconds proceeding and following 30 seconds of continuous stimulation in each patient. Gamma band power measured before stimulation was compared to gamma band power after stimulation. A) Spectral differences in all patients from channel four in the Left hemisphere. No subjects showed acute changes in gamma power. B) Spectral difference measured in the right hemisphere. TS1, TS3, and TS4 showed significant increases in gamma power following acute stimulation. Asterisk indicates significance at the 0.05 level. Box indicates TS3 and TS4 as best responders.

Since gamma played a major role in the overall clinical benefit, we investigated the role of stimulation on the short-term neuronal responses in the CM thalamus. To do so, normalized power spectra were calculated for 1-minute recordings before and after administration of 30 seconds of continuous stimulation. Average spectra for pre-stimulation trials were then subtracted from average spectra from post-stimulation trials to compute spectral difference plots. Although chronic gamma modulation was observed bilaterally in the CM, spectral difference plots did not reveal significant short term increases in oscillatory power in the left hemisphere for any patient (Figure 5). However, statistically significant changes in right hemisphere gamma power were observed in 3/5 subjects (TS1, TS3 and TS4) immediately following the stimulation event (Student’s t-test, α = 0.05). Although this change was present in all channels, it was greatest in channel four for all subjects. Channel four represents activity across the greatest possible lead distribution (across contacts 1–4) and therefore the largest spatial sampling area. Notably, no significant change in power was observed for any other frequency bands.

## Discussion

The results of this study revealed that there are band specific frequencies found in thalamic LFPs that emerge after therapeutic DBS in humans. We quantified increases in both the theta and gamma oscillations and their correlations with improvement of TS symptomatology and found that these frequency bands do not contribute equally. Those subjects that obtained the best clinical outcome from thalamic DBS exhibited the greatest gamma power changes, whereas changes in theta independently did not reveal a primary benefit. Subjects who did not exhibit gamma presence or significant increases in gamma power derived less symptomatic relief than those who exhibited gamma modulation. Thus, the correlation between gamma band activity and YGTSS in all five subjects supports the theory that thalamic gamma band dynamics are correlated with symptomatology in TS. The evidence for this is revealed upon examination of the six month difference in physiological and behavioral metrics. The two subjects who exhibited large increases in gamma also experienced greatly improved symptomatology; similarly, the two subjects who showed small changes in gamma likewise exhibited small changes in YGTSS. The most compelling demonstration of this correlation is illustrated in the dynamic changes of these parameters in TS2. As shown in Figure 4, clinical metrics and gamma power drastically change between months four and six, revealing the co-variant relationship. This correlation between gamma physiology and symptomatology raises the possibility of a functional connection between the two.

This study is the first longitudinal investigation of the pathophysiology of human TS using invasive electrophysiological methods. The study of DBS effects in TS is a nascent field and only one other study to date has investigated thalamic LFP physiology post-operatively, albeit very short term [48]. We provide here the first evidence that DBS-mediated enhancement of gamma synchronization is associated with symptom relief in TS. This inaugural cohort of 5 subjects demonstrates both the efficacy of DBS as well as state changes in the thalamic network reflected in the strength of gamma oscillations. Furthermore, the nonlinear interaction between clinical benefit and gamma precludes a surgical “stun effect”. The changes reported here occurred over many months and thus reflected a true tracking of the long term temporal dynamics of neuronal populations in response to DBS. As such, these initial results could provide direction for future studies in the critical oscillatory frequency associated with the positive symptoms of TS.

The effect of short-term stimulation on the power of these gamma oscillations mirrors the effect of long-term scheduled DBS in the CM region. This is supported by the observation that our two best responders to DBS similarly showed the largest response to short-term stimulation. A key factor to attaining therapeutic benefit from DBS is target selection, and optimal implantation locations in TS are still under debate [17], [26]. In our subjects, we experienced a spectrum of clinical benefits that we believe is due in part to variation in final lead location. Variation was observed in the final lead positions for TS1 and TS5, with TS1 lying more anterior and TS5 lying more ventral to the rest of the cohort. To control for this type of variability, future studies may evaluate an individual target region’s response to acute stimulation and use this evaluation as a marker for putative surgical targeting sites. Our results demonstrate symptom relief correlated with increased synchronization within a single oscillatory frequency. That frequency represents integration of highly circumscribed networks and therefore responses to acute stimulation could guide optimal electrode placement quickly and precisely.

Both the short-term and long-term modulations motivate deeper study on the relationship between gamma oscillations and tic expression. In general, brain oscillations arise from synchronized neuronal activity and are implicated in information coding [49]–[53]. Fast frequency rhythms in particular have been linked to functions involving tic generation, including motor preparation and stimulus selection [54]–[59]. We are interested in understanding how the changes we have reported here contribute to network dysfunction in TS [2], [10], [12], [45]. The CM thalamus receives information from both the networks of the basal ganglia as well as motor and limbic neocortex. These circuits contribute to the direct and indirect pathways that could regulate the multiple symptoms of TS. One model of TS proposes that a subset of neurons in the striatum exhibit aberrant behavior that may functionally inhibit pallidal neurons and have subsequent downstream effects throughout this circuit [60]. The function of these neuronal networks has been hypothesized to select for competing motor behaviors. In addition to the activity of these subcortical networks, cortical interaction plays a key role in motor planning and execution. Therefore, a striato-pallido-thalamo-cortical feedback loop may also be involved in the generation and propagation of tic behaviors [2], [40]. Regardless, any aberration along the network pathways, (as well as dysfunction within the thalamus itself) converges upon the CM. Synchronized gamma oscillations are a fundamental property of thalamocortical communication [61]–[63] and DBS therapy may be re-establishing normal gamma activity that is lacking in the disease state.

The novel experimental framework and results presented here provide better insight into the development of therapeutic DBS models for TS. At its core, the approach enables direct translation of neurophysiological modulations to clinical outcomes. It is possible that the identification of additional spectral features that are correlated with tic expression may be used to develop next generation responsive stimulation paradigms and they may be able to be extended to other disorders that involve intermittent and time resolved neuromodulation that is directly related to behavior. While such closed-loop DBS therapies must be tailored to individual behaviors and tics, the results from this study provide the first critical step in understanding the spectral features of LFPs that may encode tic information.

## Supporting Information

Figure S1 **Representative CT-MRI fusion showing lead localization in the CM region of the thalamus.** CM thalamus is indicated by the red arrow. Thalamus is outlined in green, striatum in blue, STN in black, and electrode trajectory is represented as a dotted red line.
(TIF)

Table S1 **Final lead locations in all subjects.**
(DOCX)

Appendix S1 **Supporting information.**
(DOCX)
