# Correction: Increased Thalamic Gamma Band Activity Correlates with Symptom Relief following Deep Brain Stimulation in Humans with Tourette’s Syndrome

**DOI:** 10.1371/annotation/446ec4cb-63da-42d2-afc6-7e8459b2abbe

**Published:** 2012-11-01

**Authors:** Nicholas Maling, Rowshanak Hashemiyoon, Kelly D. Foote, Michael S. Okun, Justin C. Sanchez

The affiliation for the first author was incorrect. Nicholas Maling is not affiliated with #1 but with #5 Department of Neurology, University of Florida, Gainesville, Florida, United States of America, and #3 Department of Neurosurgery, University of Florida, Gainesville, Florida, United States of America.

