# Supplementary figures and images for: Increased Thalamic Gamma Band Activity Correlates with Symptom Relief following Deep Brain Stimulation in Humans with Tourette’s Syndrome
Source: PLoS One. 2012 Sep 6;7(9):e44215. doi: 10.1371/journal.pone.0044215 (PMC3435399; doi:10.1371/journal.pone.0044215)

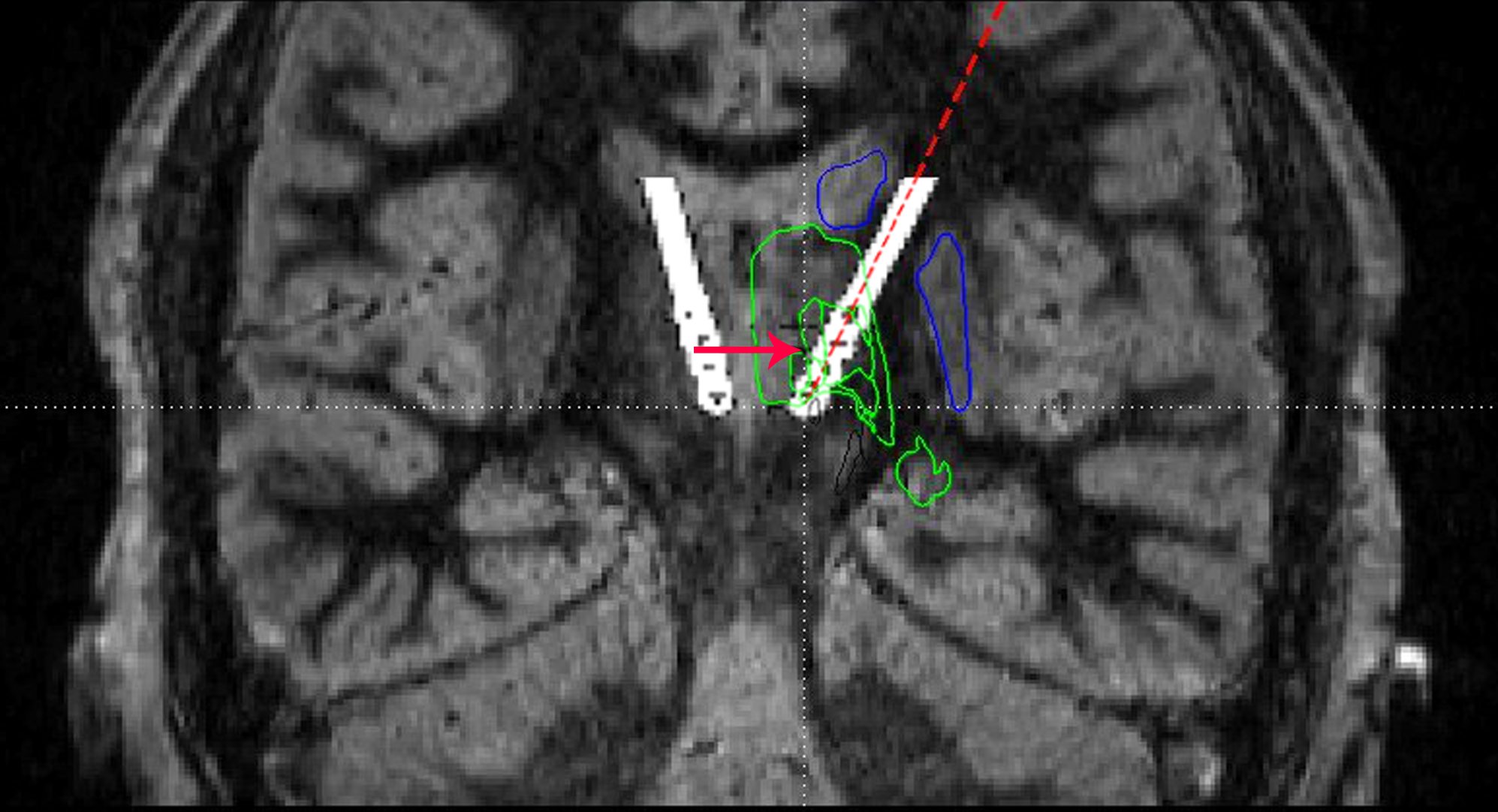

Supplement: Figure S1 — Representative CT-MRI fusion showing lead localization in the CM region of the thalamus. CM thalamus is indicated by the red arrow. Thalamus is outlined in green, striatum in blue, STN in black, and electrode trajectory is represented as a dotted red line. (TIF) [file pone.0044215.s001.tif]
