# Supplementary material for: Increased Thalamic Gamma Band Activity Correlates with Symptom Relief following Deep Brain Stimulation in Humans with Tourette’s Syndrome
Source: PLoS One. 2012 Sep 6;7(9):e44215. doi: 10.1371/journal.pone.0044215 (PMC3435399; doi:10.1371/journal.pone.0044215)
Supplement: Table S1 — Final lead locations in all subjects. (DOCX) [file pone.0044215.s002.docx]

| *Table S1. Final lead locations in all subjects* | | | | | | | | | | | | | | |
| --- | --- | --- | --- | --- | --- | --- | --- | --- | --- | --- | --- | --- | --- | --- |
|  |  |  | **Electrode 1** | | | **Electrode 2** | | | **Electrode 3** | | | **Electrode 4** | | |
| **Subject** | **Side** | **Target** | **(X)** | **(Y)** | **(Z)** | **(X)** | **(Y)** | **(Z)** | **(X)** | **(Y)** | **(Z)** | **(X)** | **(Y)** | **(Z)** |
| **TS1** | **Right** | **CM** | 5.25 | (-)1.5 | 0.17 | 5.73 | 0.34 | 3.02 | 7.17 | 1.5 | 5.88 | 8.12 | 3.48 | 8.73 |
|  | **Left** | **CM** | (-)5.39 | (-)2.59 | 0.70 | (-)6.48 | (-)0.95 | 3.53 | (-)7.92 | 0.61 | 6.36 | (-)9.42 | 2.45 | 9.19 |
| **TS2** | **Right** | **CM** | 6.21 | (-)5.25 | 0.12 | 7.44 | (-)3.16 | 2.90 | 8.87 | (-)1.63 | 5.52 | 10.2 | 0.06 | 8.22 |
|  | **Left** | **CM** | (-)3.74 | (-)3.52 | 1.34 | (-)6.09 | (-)1.32 | 3.9 | (-)6.55 | 0.40 | 6.49 | (-)8.19 | (-)2.25 | 9.06 |
| **TS3** | **Right** | **CM** | 3.08 | (-)5.36 | 0.26 | 4.07 | (-)3.31 | 2.91 | 5.03 | (-)1.24 | 5.57 | 6.10 | 0.75 | 8.22 |
|  | **Left** | **CM** | (-)7.72 | (-)5.51 | 0.14 | (-)9.17 | (-)3.79 | 2.63 | (-)10.6 | (-)1.86 | 5.12 | (-)12.3 | 0.06 | 7.61 |
| **TS4** | **Right** | **CM** | 3.59 | (-)4.45 | 0.13 | 4.87 | (-)2.80 | 2.91 | 6.28 | (-)1.15 | 5.69 | 7.5 | 0.61 | 8.47 |
|  | **Left** | **CM** | (-)2.62 | (-)5.73 | 0.21 | (-)3.90 | (-)3.78 | 2.87 | (-)5.24 | (-)1.95 | 5.53 | (-)6.52 | (-)0.12 | 8.19 |
| **TS5** | **Right** | **CM** | 3.87 | (-)3.51 | (-)1.60 | 5.11 | (-)1.54 | 1.00 | 6.24 | 0.47 | 3.60 | 7.55 | 2.38 | 6.21 |
|  | **Left** | **CM** | (-)6.53 | (-)4.44 | (-)0.86 | (-)8.03 | (-)2.58 | 1.72 | (-)9.40 | (-)0.71 | 4.31 | (-)10.9 | 1.22 | 6.89 |
| *Components from this table have been reproduced with permission from Okun (Archives of Neurology Express, 2012) and have been published with the original NIH supported FDA clinical trial NCT01329198 (clinicaltrials.gov). X=lateral to midline, Y=relative to the mid-commissural point, Z= axial relative to the AC-PC line.* | | | | | | | | | | | | | | |
