# Supplementary material for: Increased Thalamic Gamma Band Activity Correlates with Symptom Relief following Deep Brain Stimulation in Humans with Tourette’s Syndrome
Source: PLoS One. 2012 Sep 6;7(9):e44215. doi: 10.1371/journal.pone.0044215 (PMC3435399; doi:10.1371/journal.pone.0044215)
Supplement: Appendix S1 — Supporting information. (DOCX) [file pone.0044215.s003.docx]

**Supporting Information**

*Lead Localization*

A CT scan was performed one month post-operatively and fused to the pre-operative MRI to confirm lead placement (Figure S1). Lead contacts were localized to the CM region of the thalamus with the most distal contact located approximately 3mm ventral to the target region. Lead spacing was 3.5mm (center to center), resulting in contacts 1, 2 and 3 lying within the general target region and contact 4 lying dorsal (Table S1). The leads for subject TS1 were found to lie slightly more anterior than the rest of the cohort; the leads for subject TS5 were found to lie more ventral. The position in the X coordinate is measured medial/lateral relative to midline, Y is anterior/posterior relative to mid-commissural point, and Z is dorsal/ventral relative to the commissural plane.

*Signal analysis*

All offline analysis was performed on LFP recordings using MATLAB 7.10 software (MathWorks, Natick, MA, USA). Spectral power was estimated using Welch's method (1024 point hamming window, 50% overlap, 4096 point Fourier transform). For the purposes of quantitative analysis, frequency cutoffs for theta and gamma were defined as 4-7Hz and 25-45Hz, respectively. Gamma frequency was determined as optimal power fluctuations within the normal gamma range [[1-5](#_ENREF_1)]. Power values were normalized across visits for each subject by setting the sum of all power spectra to unity [[6](#_ENREF_6)]. A scaling factor of 500 was then applied to all normalized spectra such that mean gamma values were set between zero and one. Mean power was computed for all frequency bands as the average power between cutoff frequencies. Because normalization could cause frequency bias if one frequency band dominates the spectra, comparisons between theta and gamma utilized peak power values calculated from the pre-normalized data.

For analysis of chronic and acute DBS effects on thalamic activity, spectra were calculated for baseline recordings for each subject for each monthly visit. Power of the frequency bands in spectra of the chronic recordings were then correlated with clinical metrics which assessed changes in motor tic severity, phonic tic severity and overall impairment in the prior month. To examine the short term effects of stimulation on thalamic oscillations, spectral analysis was performed on recordings from the acute stimulation condition. Power spectra were calculated and combined for all the pre-stimulation recordings and also for all of the post-stimulation recordings across visits for each subject. Spectral differences were then calculated by subtracting combined pre-stimulation from combined post-stimulation spectra.

1. Cardin JA, Carlen M, Meletis K, Knoblich U, Zhang F, et al. (2009) Driving fast-spiking cells induces gamma rhythm and controls sensory responses. Nature 459: 663-667.

2. Hughes JR (2008) Gamma, fast, and ultrafast waves of the brain: their relationships with epilepsy and behavior. Epilepsy Behav 13: 25-31.

3. Ribary U (2005) Dynamics of thalamo-cortical network oscillations and human perception. Prog Brain Res 150: 127-142.

4. Llinas R, Leznik E, Urbano FJ (2002) Temporal binding via cortical coincidence detection of specific and nonspecific thalamocortical inputs: a voltage-dependent dye-imaging study in mouse brain slices. Proc Natl Acad Sci USA 99: 449-454.

5. Joliot M, Ribary U, Llinas R (1994) Human oscillatory brain activity near 40 Hz coexists with cognitive temporal binding. Proc Natl Acad Sci U S A 91: 11748-11751.

6. Liu J, Newsome WT (2006) Local field potential in cortical area MT: stimulus tuning and behavioral correlations. J Neurosci 26: 7779-7790.
